# Investigating the relationship between postoperative radiotherapy and intestinal flora in rectal cancer patients: a study on efficacy and radiation enteritis

**DOI:** 10.3389/fonc.2024.1408436

**Published:** 2024-06-26

**Authors:** Lin Long, Yexi Zhang, Jianhua Zang, Peng Liu, Wei Liu, Cheng Sun, Dan Tian, Ping Li, Jin Tian, Jun Xiao

**Affiliations:** ^1^ Qingdao Hiser Hospital Affiliated of Qingdao University (Qingdao Traditional Chinese Medicine Hospital), Qingdao, Shandong, China; ^2^ Department of Hepatobiliary Surgery, Third Affiliated Hospital of Naval Medical University, Shanghai, China; ^3^ Department of Endocrinology, Qingdao Endocrine and Diabetes Hospital, Qingdao, Shandong, China

**Keywords:** radiation enteritis, intestinal flora, fectal malignancy, radiation therapy, 16S rRNA

## Abstract

**Objective:**

This study aimed to investigate the impact of radiation therapy and radiation enteritis on intestinal flora, providing insights for treatment and prevention.

**Methods:**

Fecal samples were collected from 16 patients undergoing pelvic radiotherapy at Qingdao Hiser Hospital Affiliated of Qingdao University (Qingdao Traditional Chinese Medicine Hospital). Samples were collected before and after radiotherapy (27–30Gy), and analyzed using DNA sequencing and biostatistical methods.

**Results:**

Patients with radiation enteritis showed increased α-diversity and β-diversity of intestinal flora compared to those without radiation enteritis. Differences in flora composition were observed, with higher abundance of secondary pathways such as amino acid metabolism, carbohydrate metabolism, cofactors and vitamins metabolism, and lipid metabolism.

**Conclusion:**

The study revealed that patients developing radiation enteritis during pelvic radiation therapy had increased diversity and abundance of intestinal flora compared to those who did not develop radiation enteritis. Additionally, patients without radiation enteritis showed significantly higher diversity and abundance of intestinal flora post-radiation compared to pre-radiation.

## 1 Introduction

The role of intestinal flora in tumor therapy has garnered significant attention in recent years. The effectiveness of antitumor treatments, such as chemotherapy and immunotherapy, is influenced by the intestinal flora (1). Radiotherapy is a crucial component of cancer treatment, with over 50% of cancer patients undergoing radiotherapy at some point. Approximately 60% of these patients derive benefits from radiotherapy (2), although its efficacy and potential adverse effects are influenced by various factors. The relationship between gut flora and radiotherapy is bidirectional; radiotherapy can disrupt gut flora, while changes in gut flora can impact the efficacy of radiotherapy. Shiao et al. (3) discovered that antibiotic-induced bacterial depletion in mouse models of breast cancer and melanoma hindered the activation of macrophages and CD8+ T-cells, thus limiting the effectiveness of radiotherapy. Conversely, antifungal drug-induced fungal depletion reduced the presence of M2 macrophages, which have an immunosuppressive function, thereby alleviating immunosuppression during radiotherapy. Additionally, this depletion enhanced the activity of CD8+ T-cells, ultimately improving the efficacy of radiotherapy.

Radiation-induced enteritis (RE) is a prevalent side effect of pelvic radiotherapy, with over 80% of patients experiencing it during treatment (4). Clinical symptoms typically include diarrhea, abdominal pain, and rectal bleeding. In severe cases, RE can progress to life-threatening systemic infections within a short timeframe. Due to the risk of RE, treatment may involve reducing radiation doses or even halting therapy, which can significantly impact patients’ well-being and survival rates. The pathogenesis of RE is multifaceted, and current preventive and treatment options are not yet standardized or highly effective. Recent research suggests that radiotherapy can alter the composition of intestinal flora, with specific microbial populations potentially playing a role in the development of RE.

Numerous recent studies have elucidated the intricate pathways and mechanisms linking the gut-liver axis, the gut-lung axis, the gut-brain axis, and potentially the gut-cardiac axis with intestinal flora and various associated diseases (5–7). These findings underscore the significance of investigating intestinal flora and emphasize the crucial need to maintain beneficial interactions between humans and their associated intestinal flora for optimal health. Research has indicated that patients with radiation enteritis (RE) exhibit an imbalance in intestinal flora, characterized by a reduction in beneficial bacteria and an increase in pathogenic bacteria, which may exacerbate radiation-induced intestinal damage (8). The advent of 16S rRNA sequencing technology, known for its cost-effectiveness and efficiency in analyzing microbial communities across numerous samples, has shed light on the intimate connection between intestinal flora and various diseases. However, limited studies have explored the relationship between radiation enterocolitis and intestinal flora, with inconsistent conclusions. This study aims to investigate the correlation between radiation enteritis and intestinal flora, alterations in intestinal flora following pelvic radiotherapy, and the role of specific flora in metabolic pathways. The findings of this research are expected to offer valuable insights for developing personalized radiation therapy plans and strategies for preventing and treating radiation enteritis in patients.

## 2 Research content and methodology

### 2.1 Study subjects

Sixteen patients who were admitted to Qingdao Hiser Hospital Affiliated of Qingdao University (Qingdao Traditional Chinese Medicine Hospital) for postoperative adjuvant radiotherapy for rectal cancer between November 2021 and November 2023 were included in the study. All participants had a clear understanding of the experimental procedures and had signed the Informed Consent Form. The study was approved by the Ethics Committee of Qingdao Hiser Hospital Affiliated of Qingdao University (Qingdao Traditional Chinese Medicine Hospital). Ethical Approval Number:2021HC01LS022.

#### 2.1.1 Inclusion criteria

Patients over 18 years old with a confirmed pathological diagnosis of stage II/III rectal malignant tumors undergoing postoperative adjuvant radiotherapy were included in the study. None of the patients had undergone radiotherapy or any other antitumor therapy prior to surgery. The experimental group consisted of patients with a grade 2 radiological reaction, while the control group consisted of patients with a grade 0 radiological reaction. All patients received conformal intensity-modulated radiotherapy (IMRT) for the first time, with a radiotherapy dose ranging from 1.8–2.0Gy over 45–50Gy. Informed consent was obtained from all participants.

#### 2.1.2 Exclusion criteria

Patients excluded from this study included those with gastrointestinal diseases not related to radiotherapy, such as acute or chronic intestinal obstruction, Crohn’s disease, ulcerative colitis, intestinal tuberculosis, bacillary dysentery, etc.; those with second primary non-abdominopelvic malignant tumors; those with severe cardiac, hepatic, renal, cerebral, and other organ dysfunctions; those taking medications such as antibiotics, gastrointestinal bacterial flora modifiers, gastric acid suppression, immunosuppression, etc., within the past month; those with a history of human immunodeficiency virus, active syphilis, active pulmonary tuberculosis, and active hepatitis; and those with serious bone marrow suppression and organ failures.

#### 2.1.3 Diagnostic criteria

In accordance with the RE-related diagnostic criteria outlined in the Chinese Expert Consensus on Multidisciplinary Diagnosis and Treatment of Radiation Rectal Injury (2021 Edition), patients diagnosed with radiolucent enterocolitis were confirmed through clinical diagnosis. Patients with radiolucent enterocolitis were assessed for radiation response based on the RTOG and EORTC criteria. Grade 0 indicates no change, grade 1 signifies an increase in the number of stools or changes in bowel habits not requiring medication, along with rectal discomfort not necessitating pain medication. Grade 2 involves diarrhea necessitating anti-parasympathomimetic drugs, mucus evacuation without toilet paper, or rectal/abdominal pain requiring analgesics. Grade 3 includes diarrhea requiring parenteral nutritional support, bleeding, abdominal distension necessitating toilet paper (confirmed by x-ray radiographs of dilated intestinal loops), and grade 4 encompasses acute or subacute intestinal obstruction, perforation, sinusocele, bleeding requiring transfusion, and abdominal or ligamentary pain necessitating gastrointestinal decompression or intestinal rerouting.

We collected pre-radiotherapy fecal samples from 50 patients, followed by 15 radiotherapy sessions. Subsequently, we screened and enrolled 16 patients undergoing pelvic radiotherapy based on the aforementioned criteria, dividing them into two groups according to the RTOG Acute Radiological Response Scoring Criteria. The groups consisted of 8 patients in the RE group and 8 patients in the no-RE group.

### 2.2 Content and methodology

#### 2.2.1 Baseline information collection

Baseline information of 16 patients were collected: age, sex, body mass index, history of smoking, hypertension, diabetes mellitus, coronary artery disease, and laparotomy.

#### 2.2.2 Collection and preservation of fecal specimens

According to existing literature and clinical observations, radiation enteritis typically occurs after 15 to 20 sessions of radiotherapy. Therefore, patients undergoing this study provided stool specimens before radiotherapy and after 15 sessions of radiotherapy (27–30Gy). The day before starting treatment and the day after 15 treatments, patients were instructed to collect the first stool specimen in the early morning on an empty stomach. Using a small spoon, approximately one-third of the middle feces volume was collected in a special sterile tube, ensuring a specimen volume greater than 1g. Patient details including name, hospitalization number, tumor type, radiation therapy dose, and collection time were recorded on the tube. The specimen was promptly stored in a -80°C refrigerator within 2 hours and subsequently sent to Ovison Biomedical Technology Co.

#### 2.2.3 16SrRNA data analysis

Genomic DNA was extracted from fecal samples using 16S rRNA sequencing and biostatistical analysis. The highly variable regions of microbial 16S rRNA were specifically amplified to construct small fragment libraries based on the characteristics of the amplified regions. These libraries underwent double-end sequencing on the Illumina NovaSeq platform. The final Amplicon Sequence Variants (ASVs) were obtained through splicing, de-limiting, de-chimerization, and noise reduction using the DADA2 method in QIIME2 software. The valid data obtained were then subjected to sequence analysis and species annotation to determine the species composition of the samples. Alpha diversity was assessed using the Chao1 index for colony abundance, Shannon index, and Simpson index for colony diversity. Beta diversity was assessed using Partial Least Squares Discriminant Analysis (PLS-DA). Species differences were analyzed, and marker species were identified using LEfSe (LDA Effect Size) analysis and Metastats analysis (9). The assay analysis was conducted by Ovison Biomedical Technology Co.

#### 2.2.4 Statistical methods

Using SPSS 26.0 statistical analysis software, the data were analyzed. Normally distributed quantitative variables were described using mean ± standard deviation. The comparison of the two groups was conducted using t-test. For non-normally distributed data, the M (P25, P75) was used to describe the data, and the comparison of the two groups was done using the rank-sum test. Qualitative variables were presented as number and proportion (%), and group comparisons were made using chi-square test or Fisher’s exact probability method.

## 3 Results

### 3.1 Comparison of the general conditions of the two groups of patients

Among the 16 patients, there were 8 with radiation enteritis and 8 with non-radiation enteritis. The two groups consisted of 9 males and 7 females, with 6 patients having a history of smoking and 10 without. Additionally, 9 patients had hypertension while 7 did not, and 3 patients had diabetes mellitus while 13 did not. Furthermore, 5 patients had coronary artery disease and 11 did not. Statistical analysis revealed that there were no significant differences in gender, age, BMI, history of smoking, hypertension, or diabetes mellitus between the two groups (P>0.05), indicating their comparability. See **Table 1** for more details.

**Table 1:** Baseline data of 16 radiotherapy patients.

			RE	NO RE	statistic	*P*
**genders**	Male	9	5	4	0.254	0.614
	Female	7	3	4		
**age**	57.312 ± 8.942		56.375 ± 3.145	58.250 ± 3.358	-0.408	0.690
**BMI**	25.5 ± 3.011		25.625 ± 1.179	25.375 ± 1.017	0.161	0.875
**history of smoking**	Yes	6	3	3	0.000	1.00
	No	10	5	5		
**hypertension**	Yes	9	4	5	0.254	0.614
	No	7	4	3		
**diabetes**	Yes	3	2	1	0.410	0.522
	No	13	6	7		
**Coronary artery disease**	Yes	5	2	3	0.291	0.590
	No	11	6	5		

### 3.2 Sequencing data analysis

**Figure 1:**
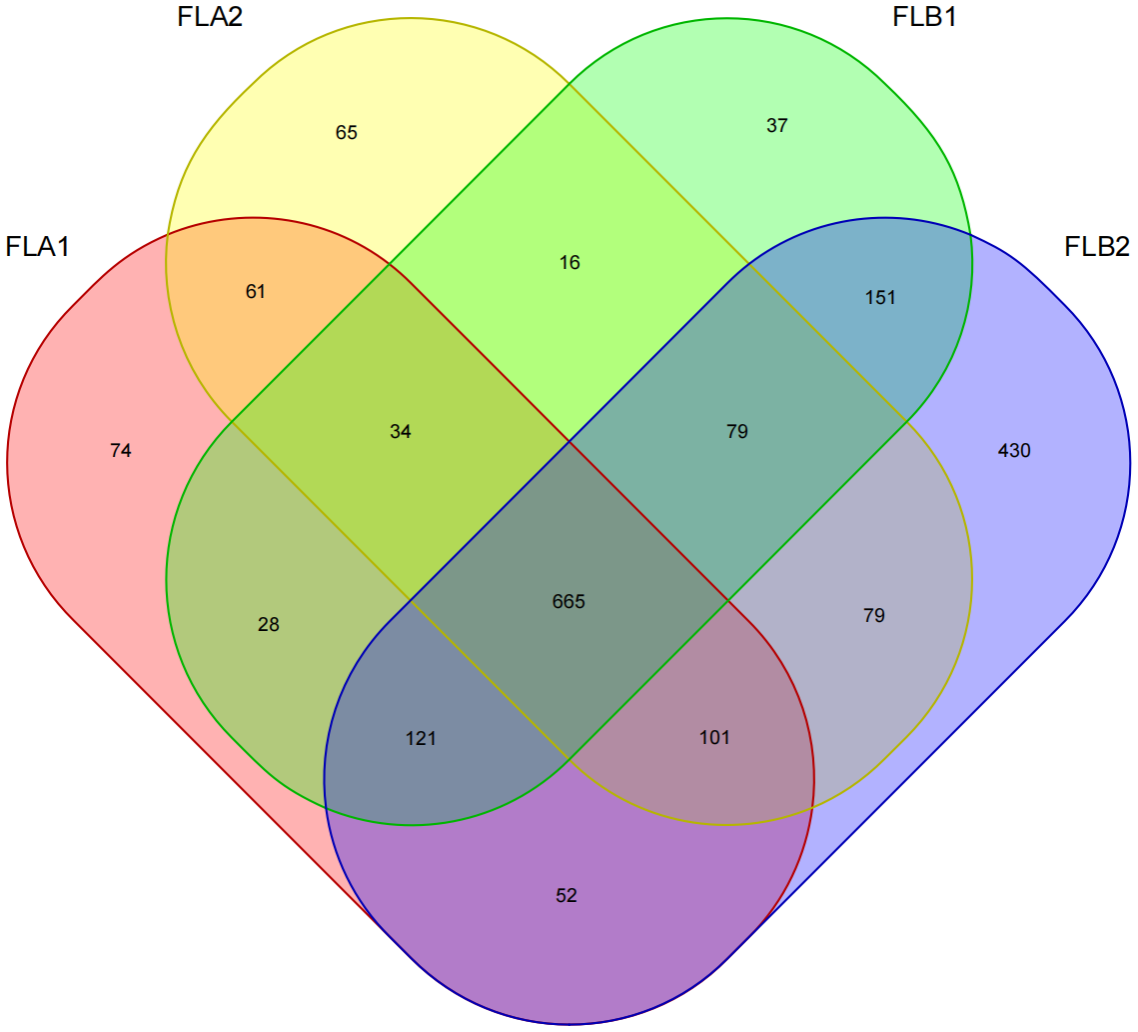
Wayne plots, number of OTUs for four sample groups.

**Figure 2:**
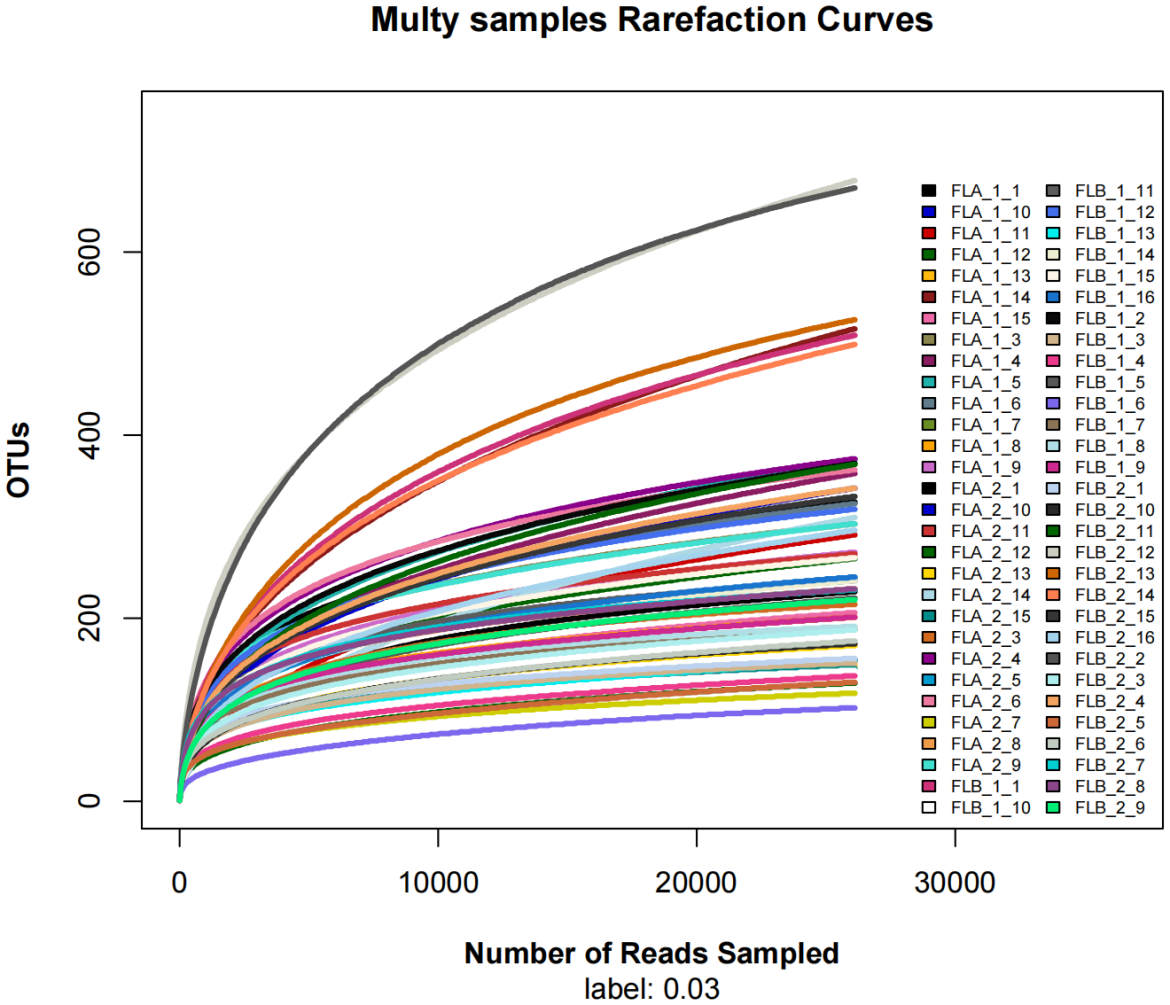
Dilution curves, the sequences of each sample tend to be stable (horizontal coordinate: amount of randomly selected sequencing data; vertical coordinate: number of observed OTUs).

**Figure 3:**
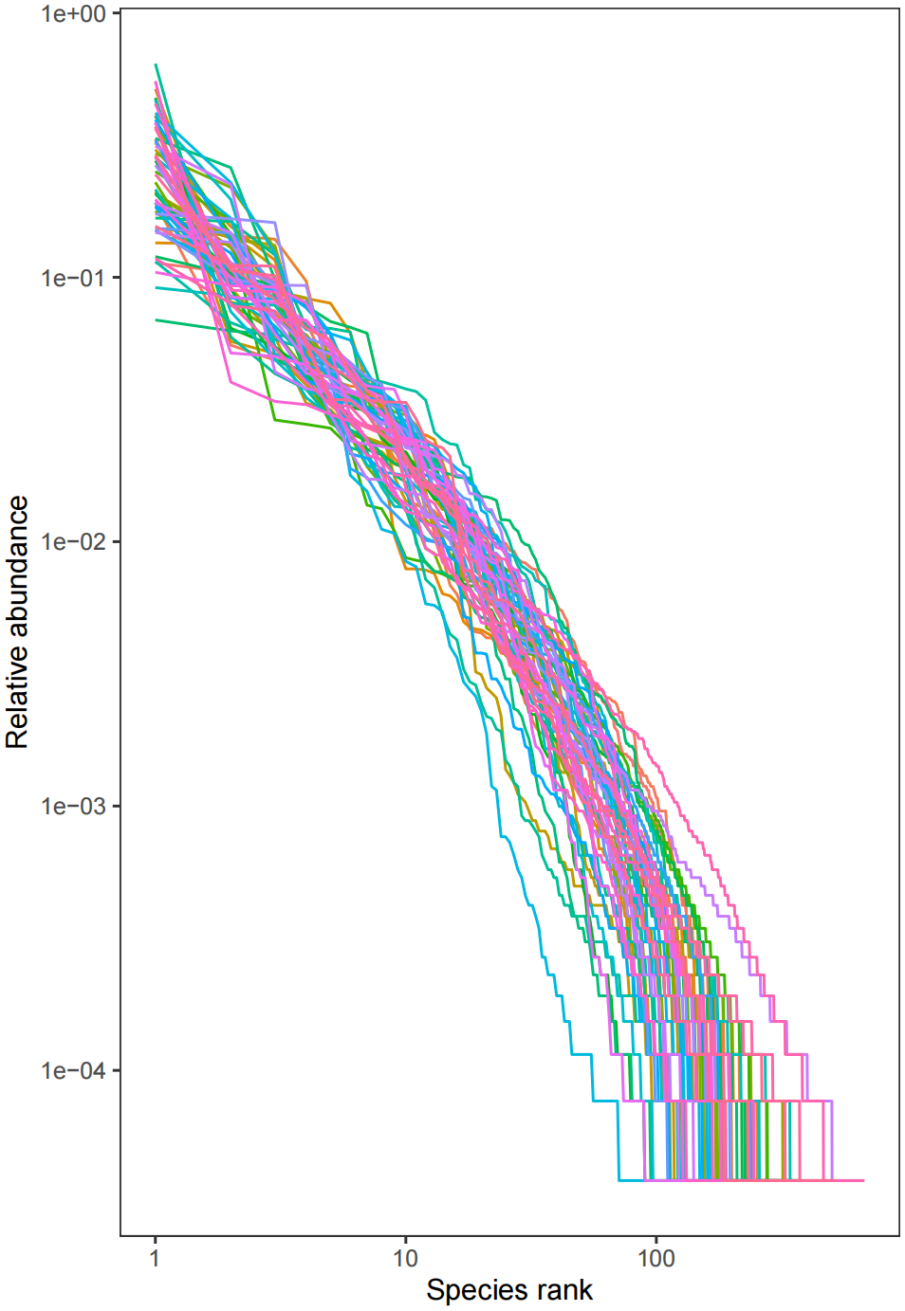
Abundance rank curve, the abundance of each sample is higher, and the fold line is smoother (horizontal coordinate: OTU rank, vertical coordinate: the relative percentage of the number of sequences in OTUs of this rank, i.e., the number of sequences belonging to this OTU is divided by the total number of sequences, the numbers on the vertical axis, e.g., “10^-2” represents the relative abundance of 0.01, “10^-1” represents the relative abundance of 0.1, and so on.). (e.g. “10^-1” represents a relative abundance of 0.1, and so on).

Sixteen patients were categorized based on the presence or absence of radiation enteritis (RE). Samples from the group with RE were labeled as FLB2, while their pre-radiotherapy samples were labeled as FLB1. The group without RE was named FLA2, with their pre-radiotherapy samples labeled as FLA1. A total of 2060 operational taxonomic units (OTUs) were identified across the four groups, with 1993 OTUs remaining after processing and filtering. The Venn diagram in **Figure 1** illustrated that 1,387 OTUs were shared among all groups, with 74 OTUs specific to FLA1 and 65 specific to FLA2. The FLA1 group had 74 unique OTUs, FLA2 had 65, FLB1 had 37, and FLB2 had 430, indicating a higher abundance of unique bacteria in the RE group. The number of OTUs in the RE group was significantly greater than in the non-RE group, reflecting notable changes in the intestinal flora due to radiation therapy-induced radiation enteritis. Dilution curves for each sample showed saturation with increased sequencing, indicating sufficient sequencing depth to capture the diversity within all samples (**Figure 2**). Abundance rank abundance curves displayed a relatively flat distribution pattern across all four groups, suggesting low variability in flora abundance and high uniformity among test samples (**Figure 3**). These results suggest that the population samples and sequencing depth in this study meet high standards and demonstrate high reliability.

### 3.3 Relationship of intestinal flora diversity to radiation enteritis and radiotherapy

**Figure 4:**
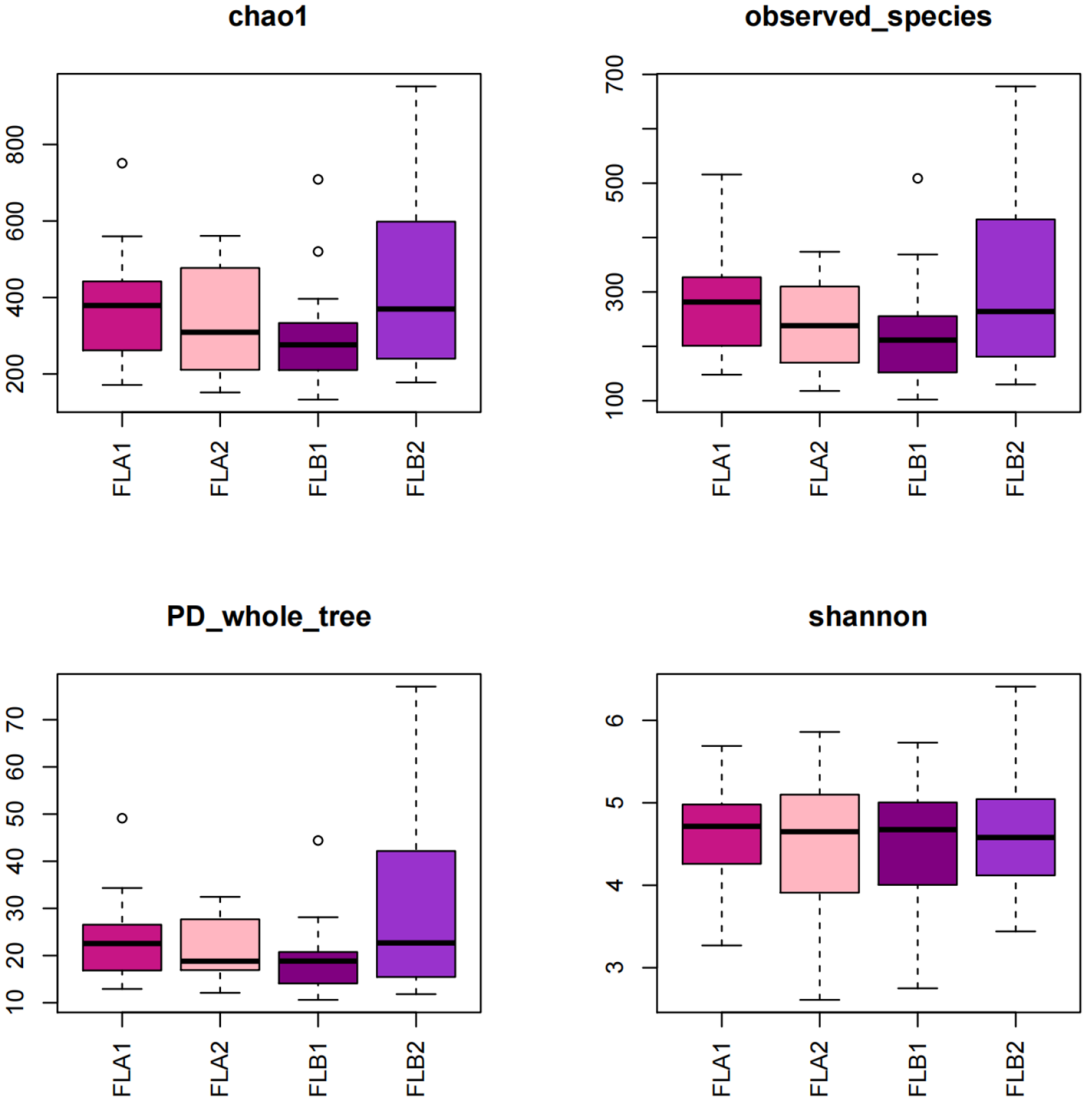
Box plot of alpha diversity index of intestinal flora.

The study compared the α-diversity and β-diversity of intestinal flora in two groups before and after radiotherapy with equal irradiation. Under the same irradiation dose, the Tukey test revealed that the Chao1 index and the PD_whole_tree index of the FLB2 group was significantly higher than that of the FLA1 and FLB1 groups (*P* < 0.05). Additionally, the observed_species index of the FLB2 group was significantly higher than that of the FBL1 group (*P* < 0.05). These differences were statistically significant, while the Shannon index did not show any statistical differences across all four groups (**Figure 4**). These results indicate that the bacterial flora richness in patients from the RE group increased significantly after radiotherapy (*P* < 0.05), while the diversity of bacterial flora did not show significant differences (*P* > 0.05) at the same radiotherapy dose.

**Figure 5:**
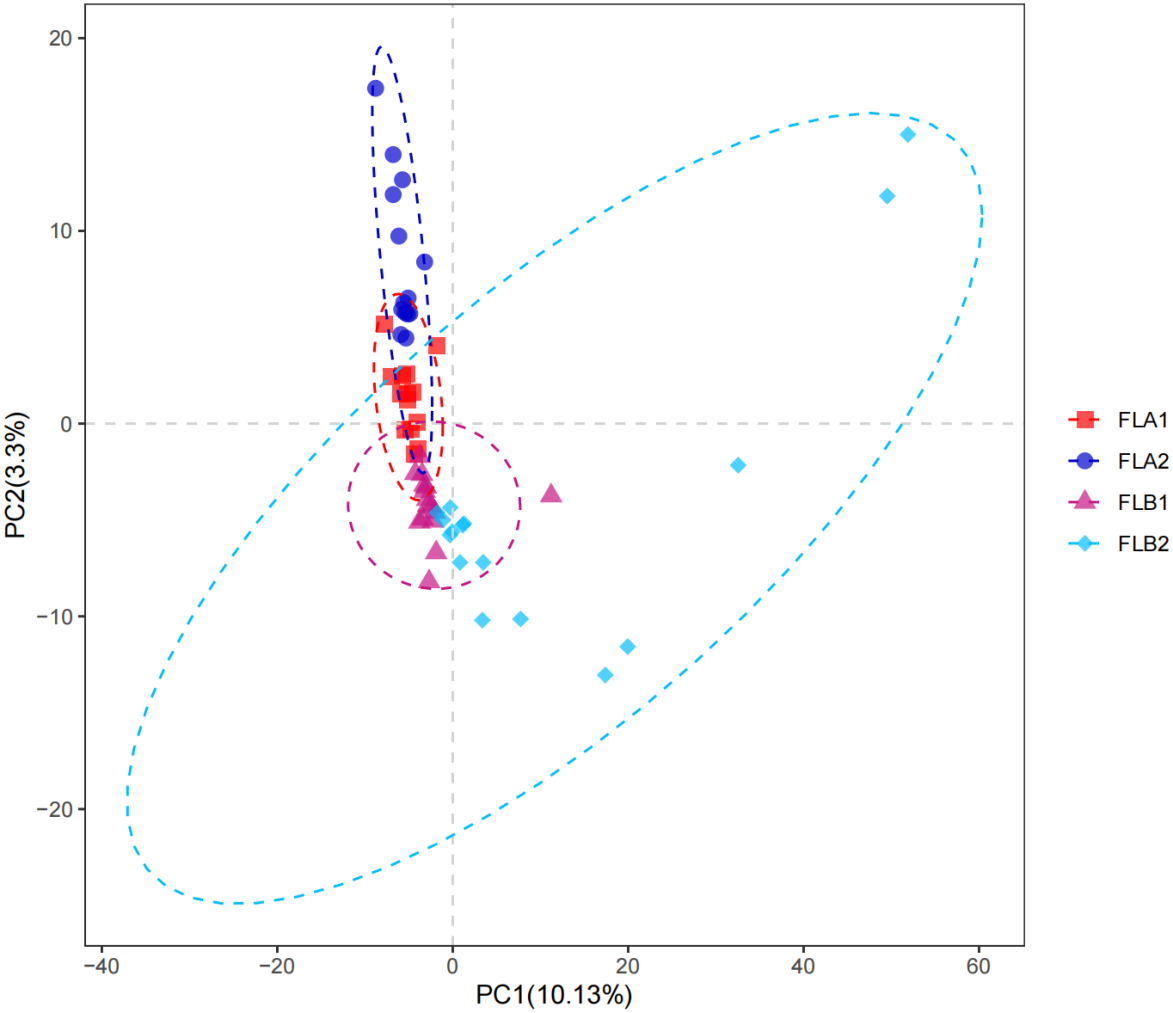
β-diversity of intestinal flora.

Microbial community intergroup variability (Beta diversity) was assessed using Partial Least Squares Discrimination Analysis (PLS-DA). The ANOSIM nonparametric test indicated a statistically significant difference between the FLB2 and FLA2 groups, with the difference between the two groups being significantly greater than the difference within the groups. The β-diversity of the bacterial flora in patients with RE was found to be higher than in those without RE (*P* < 0.05), as illustrated in **Figure 5**.

### 3.4 Differences in intestinal flora between the two groups of patients

**Figure 6:**
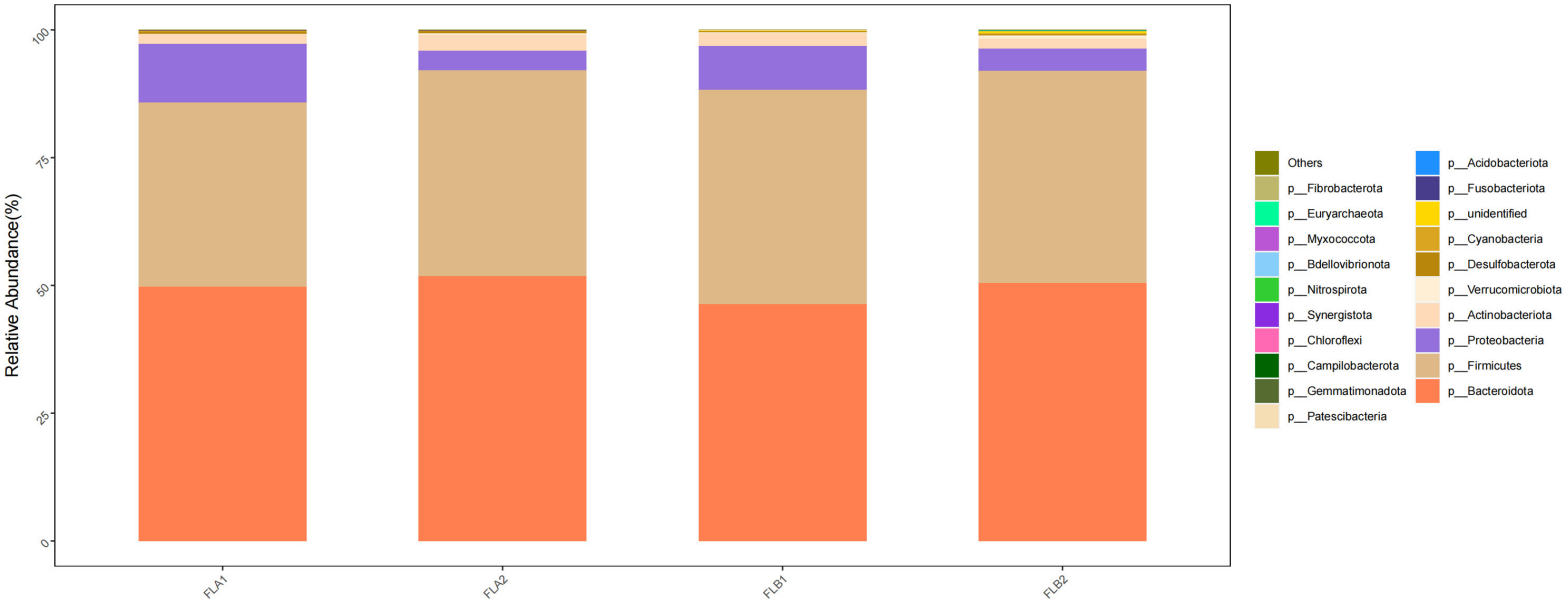
Differences in the abundance of intestinal flora at the level of phylum among the four sample groups in radiotherapy.

Based on the classification of phylum, class, order, family and genus of intestinal flora, the relative abundance of bacterial species in the two groups of patients under radiotherapy was compared at the level of different types. At the level of phylum, p:Bacteroidota and p:Firmicutes were predominant among the four groups, and the proportion of p:Bacteroidota was 50.48% and 51.90% in the FLB2 and FLA2 groups, respectively, 51.90% and 49.79% in the FLA2 and FLA1 groups; the proportions of p:Firmicutes in FLB2 and FLA2 groups were 41.54% and 40.21%, respectively, and the proportions of p:Firmicutes in FLA2 and FLA1 groups were 40.21% and 36.07%, respectively; Followed by p:Proteobacteria (4.31% and 3.87%; 3.87% and 11.38%), p:Actinobacteriota, (2.01% and 3.01%; 3.01% and 1.96%); In comparison to the non-RE group, the abundance of p:Proteobacteria and p:Firmicutes increased after radiotherapy, while p:Actinobacteriota and p:Bacteroidota decreased. Conversely, in the no-RE group, the abundance of p:Bacteroidota, p:Firmicutes, and p:Actinobacteriota increased, while p:Proteobacteria decreased post-radiotherapy. However, the results of the Kruskal-Wallis rank sum test analysis indicated that none of the observed changes in microflora abundance were statistically significant. As shown in **Figure 6**.

**Figure 7:**
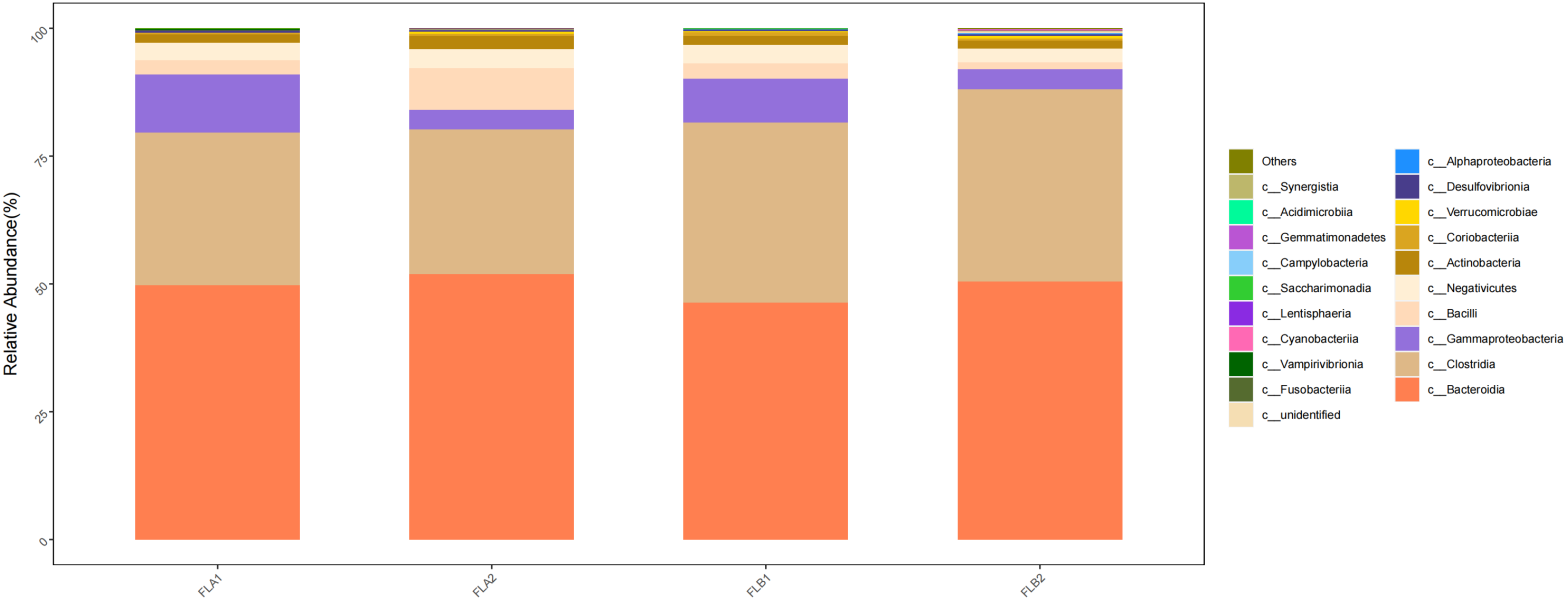
Differences in the abundance of intestinal flora at the level of class among the four sample groups in radiotherapy.

At the class level, c:Bacteroidia and c:Clostridia were dominant in the four groups, the proportion of c:Bacteroidia in the FLB2 and FLA2 groups were 50.47% and 51.90%, respectively, and the proportion in the FLA2 and FLA1 groups being 51.90% and 49.79%; c:Clostridia had 37.58% and 28.37% in FLB2 and FLA2 groups, respectively, and 28.37% and 29.88% in FLA2 and FLA1 groups, respectively; Followed by c:Gammaproteobacteria (4.31% and 3.87%; 3.87% and 11.30%), c:Bacilli (1.35% and 8.12%; 8.12% and 2.82%), and (c:Negativicutes (2.61% and 3.71%; 3.71% and 3.38%); compared to the non-RE group, the results of the Kruskal-Wallis rank sum test analysis indicated that there was a notable increase in the abundance of c:Clostridia and c:Gammaproteobacteria, while c:Bacteroidia, c:Bacilli and c:Negativicutes decreased. Conversely, in the non-RE group, c:Bacteroidia, c:Bacilli and c:Negativicutes showed an increase in abundance, while c:Clostridia and c:Gammaproteobacteria decreased. Analysis using the Kruskal-Wallis rank sum test indicated that the abundance of c:Bacilli was statistically significant in both the RE and no RE groups, whereas the abundance of other bacterial groups did not show significance As shown in **Figure 7**.

**Figure 8:**
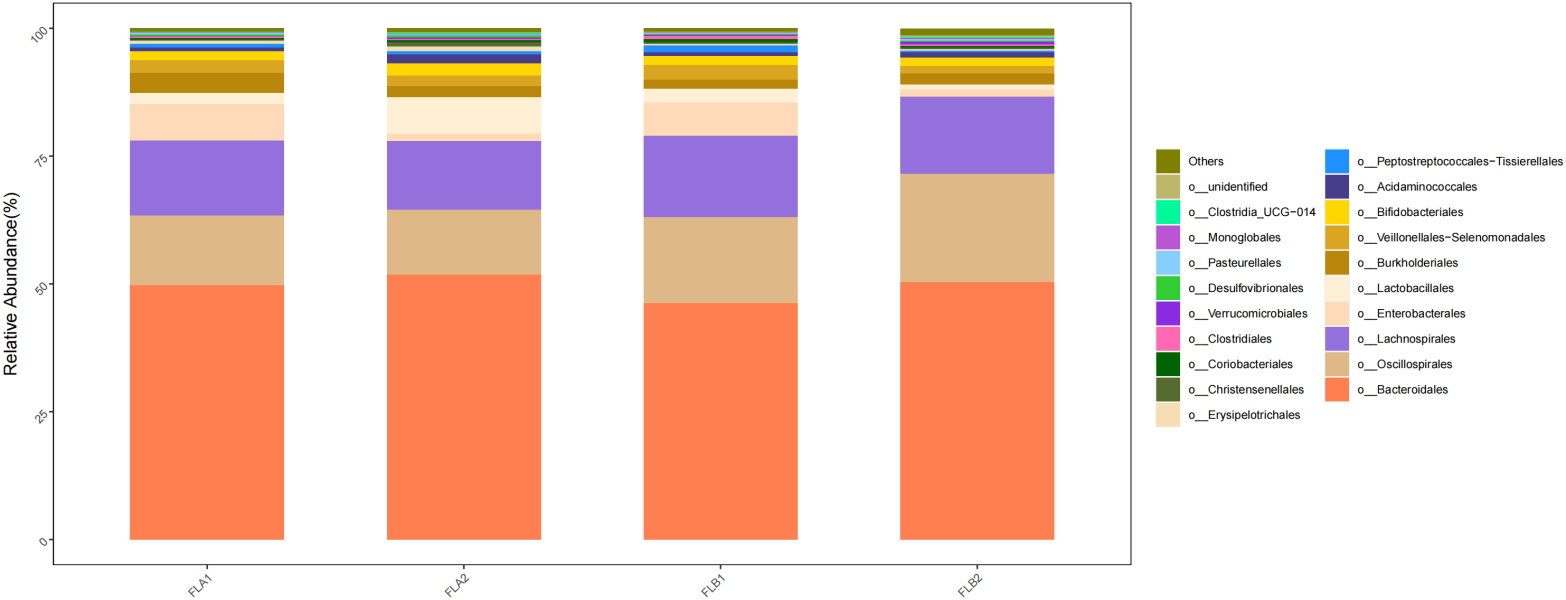
Differences in the abundance of intestinal flora at the level of the order in the four groups of samples in radiotherapy.

At the order level, the main orders were o:Bacteroidales (50.36% and 51.87%; 51.87% and 49.75%), o:Oscillospirales (21.20% and 12.64%; 12.64% and 13.67%), o: Lachnospirales (15.14% and 13.50%; 13.50% and 14.65%) and o:Burkholderiales (2.11% and 2.15%; 2.15% and 3.86%). Compared to the non-RE group, the results of the Kruskal-Wallis rank sum test analysis indicated that the abundance of o:Oscillospirales increased significantly, the difference was statistically significant, the abundance of o: Lachnospirales increased, and the abundance of o:Bacteroidales and o:Burkholderiales decreased,the difference was not statistically significant, In the non-RE group, the abundance of o:Bacteroidales increased and the abundance of o:Oscillospirales, o: Lachnospirales and o:Burkholderiales decreased in the post-radiotherapy samples,there was no statistically significant difference. As shown in **Figure 8**.

**Figure 9:**
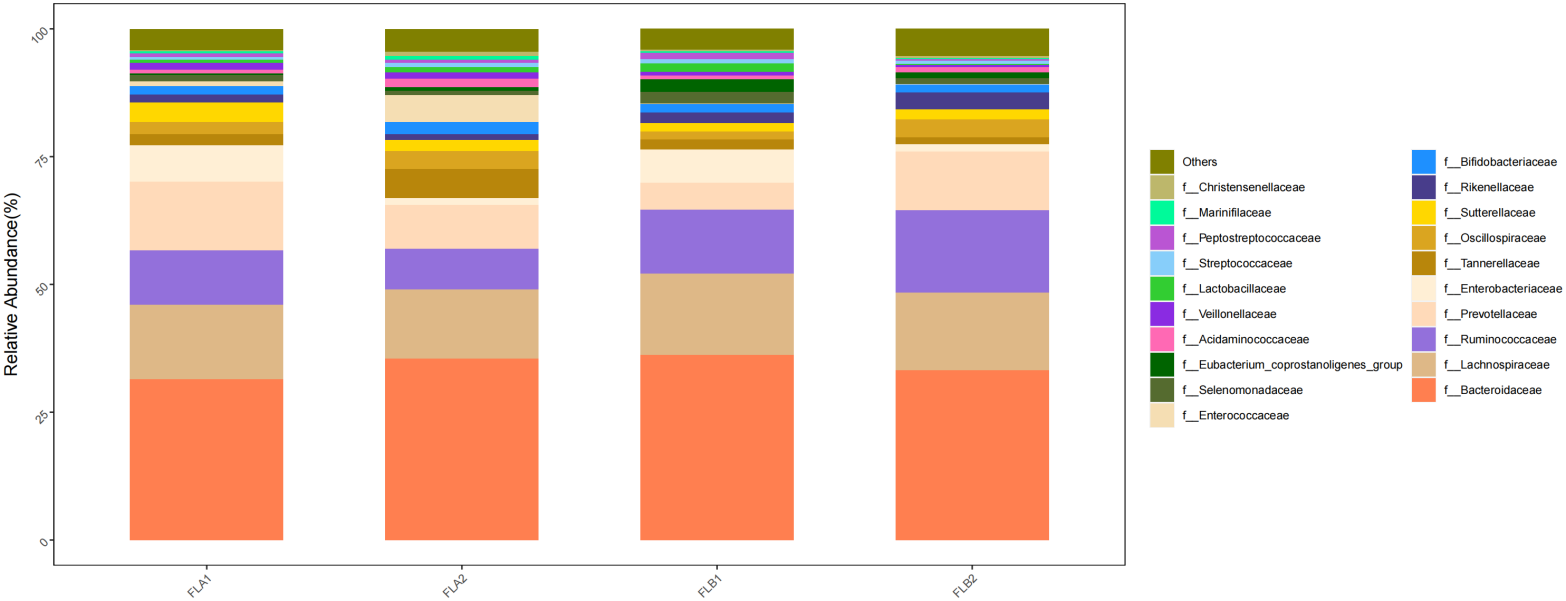
Differences in the abundance of intestinal flora at the level of the family in the four groups of samples in radiotherapy.

At the family level, the main families were f:Bacteroidaceae (33.27% and 35.50%; 35.50% and 31.43%), f:Ruminococcaceae (16.12% and 8.00%; 8.00% and 10.59%), f: Lachnospiraceae(15.14% and 13.50%; 13.50% and 14.65%), f:Prevotellaceae(11.52% and 8.52%; 8.52% and 13.41%) and f:Oscillospiraceae(3.54% and 3.50%; 3.50% and 2.33%). Compared to the non-RE group, the results of the Kruskal-Wallis rank sum test analysis indicated that the abundance of f:Ruminococcaceae increased significantly in the RE group, the difference was statistically significant, f: Lachnospiraceae, f:Prevotellaceae increased in the RE group, whereas f:Bacteroidaceae decreased,there was no statistically significant difference. f:Oscillospiraceae were of comparable abundance. In the non-RE group, the abundance of f:Bacteroidaceae and f:Oscillospiraceae increased and the abundance of f:Ruminococcaceae, f: Lachnospiraceae and f:Prevotellaceae decreased in the post-radiotherapy samples,there was no statistically significant difference. As shown in **Figure 9**.

**Figure 10:**
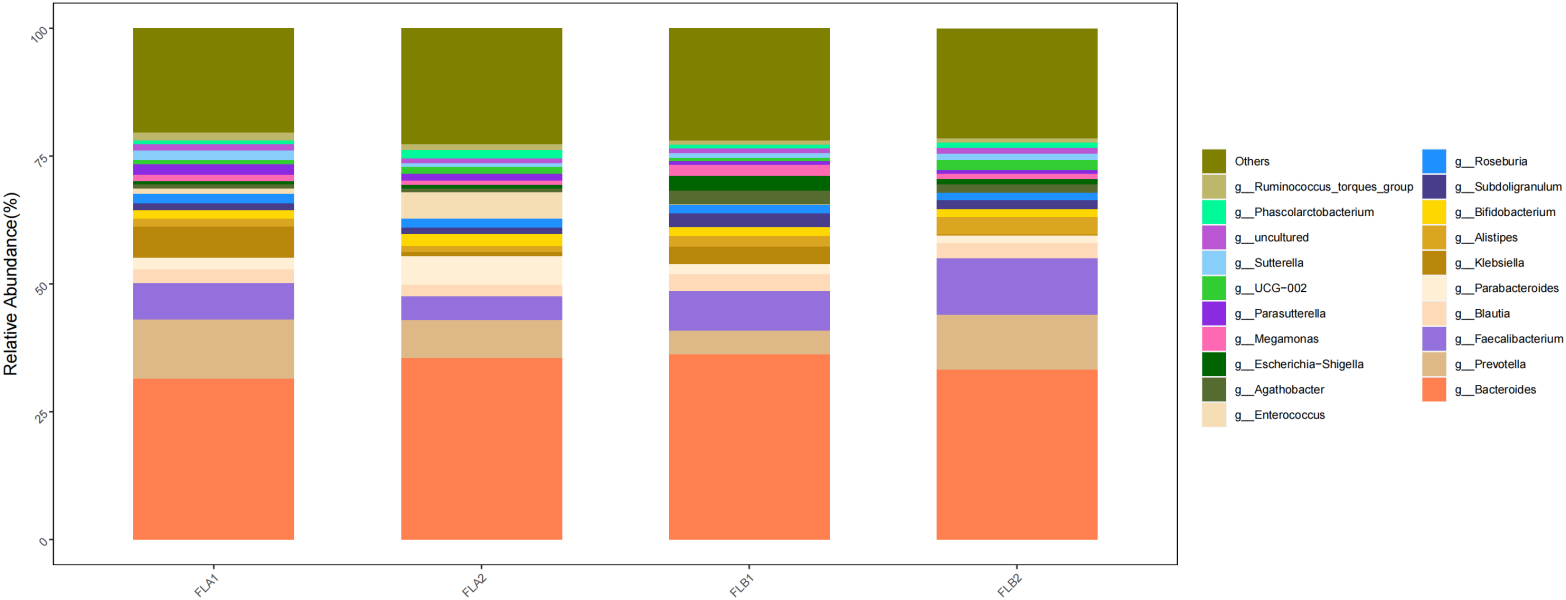
Differences in the abundance of intestinal flora at the genus level among the four groups of samples in radiation therapy.

At the genus level, the main genera were g:Bacteroides (33.27% and 35.50%; 35.50% and 31.43%), g:Faecalibacterium (11.02% and 7.50%; 7.50% and 7.05%), g:Prevotella(10.73% and 7.43%; 7.43% and 11.66%), g:Alistipes(3.35% and 1.12%; 1.12% and 1.56%) and g:Blautia(3.87% and 2.26%; 2.26% and 2.73%). Compared to the non-RE group, the results of the Kruskal-Wallis rank sum test analysis indicated that the abundance of g:Faecalibacterium increased in the RE group, the difference was statistically significant, g:Prevotella, g:Alistipes, and g:Blautia increased whereas that of g:Bacteroides decreased,there was no statistically significant difference. In the non-RE group, the abundance of B g:Bacteroides and g:Faecalibacterium increased, while the abundance of g:Prevotella, g:Alistipes and g:Blautia decreased,there was no statistically significant difference. As shown in **Figure 10**.

**Figure 11:**
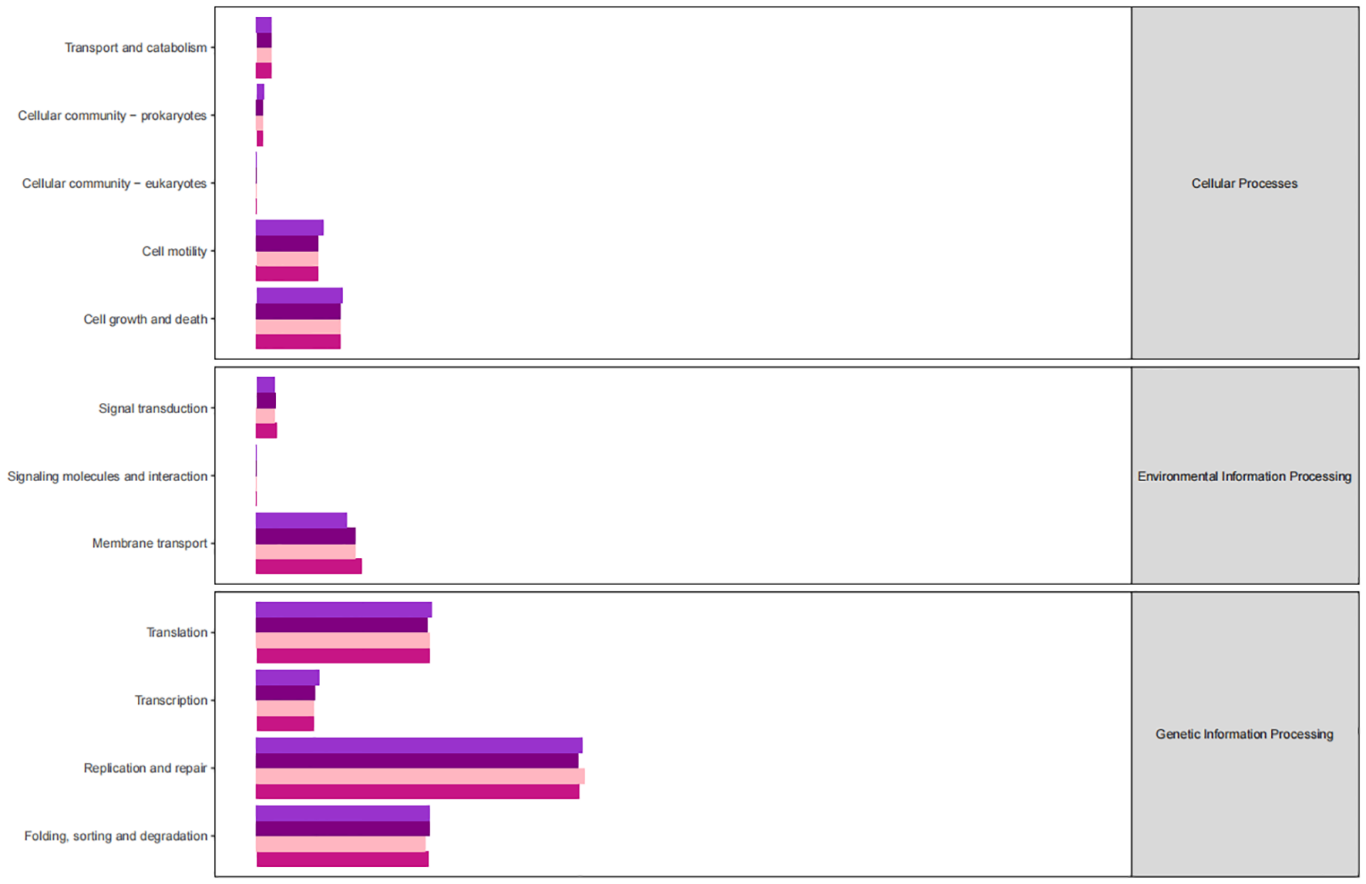
Predicted KEGG functional pathway abundance map (horizontal coordinates: abundance of functional pathways, vertical coordinates: functional pathways at the second classification level of KEGG).

**Figure 12:**
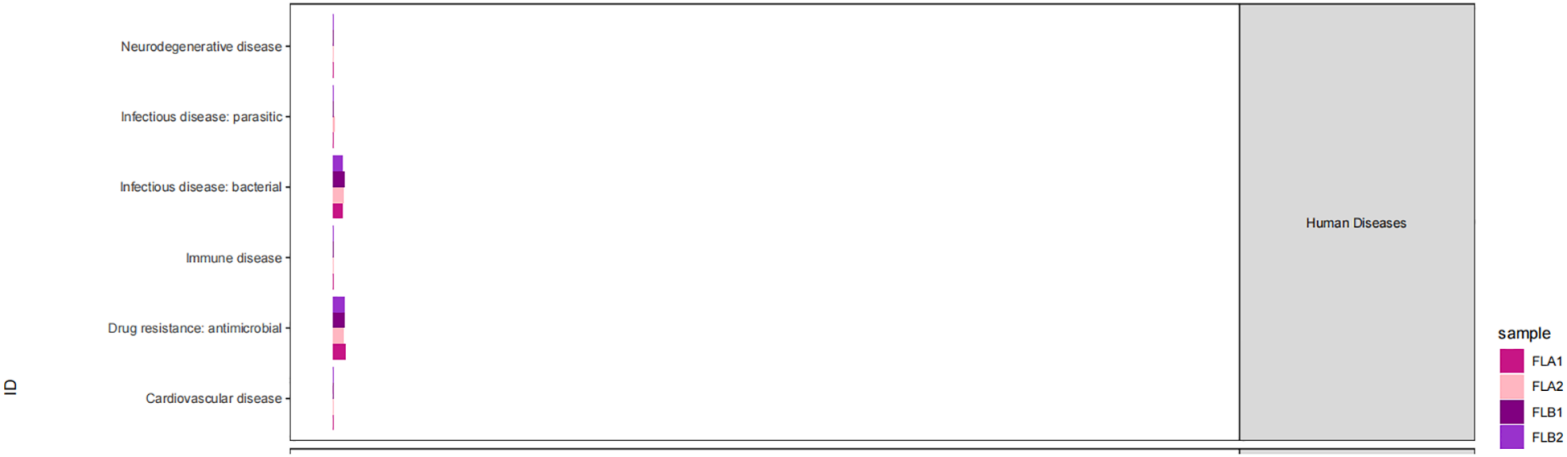
Predicted KEGG functional pathway abundance map (horizontal coordinates: abundance of functional pathways, vertical coordinates: functional pathways at the second classification level of KEGG).

**Figure 13:**
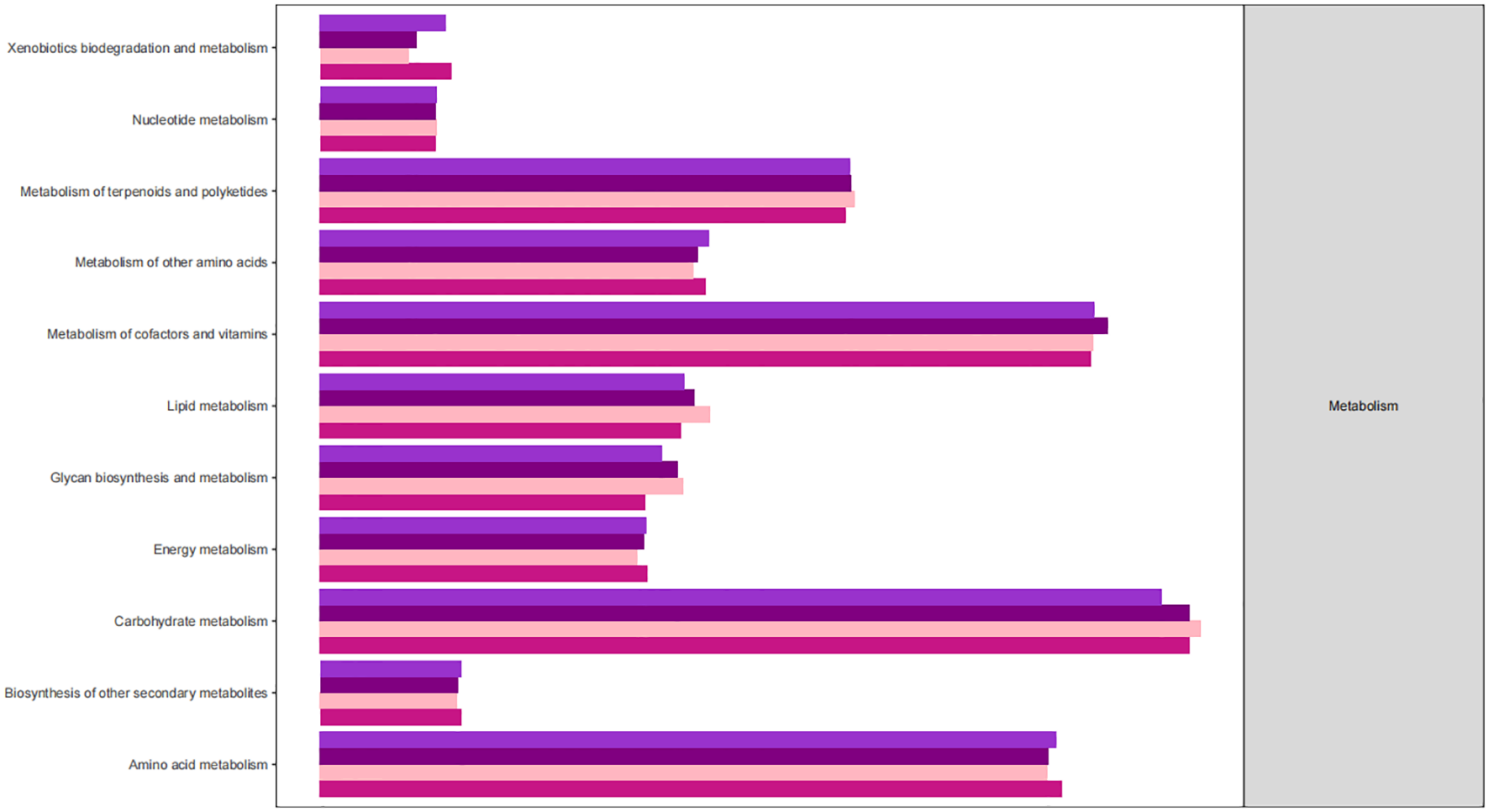
Predicted KEGG functional pathway abundance map (horizontal coordinates: abundance of functional pathways, vertical coordinates: functional pathways at the second classification level of KEGG).

**Figure 14:**
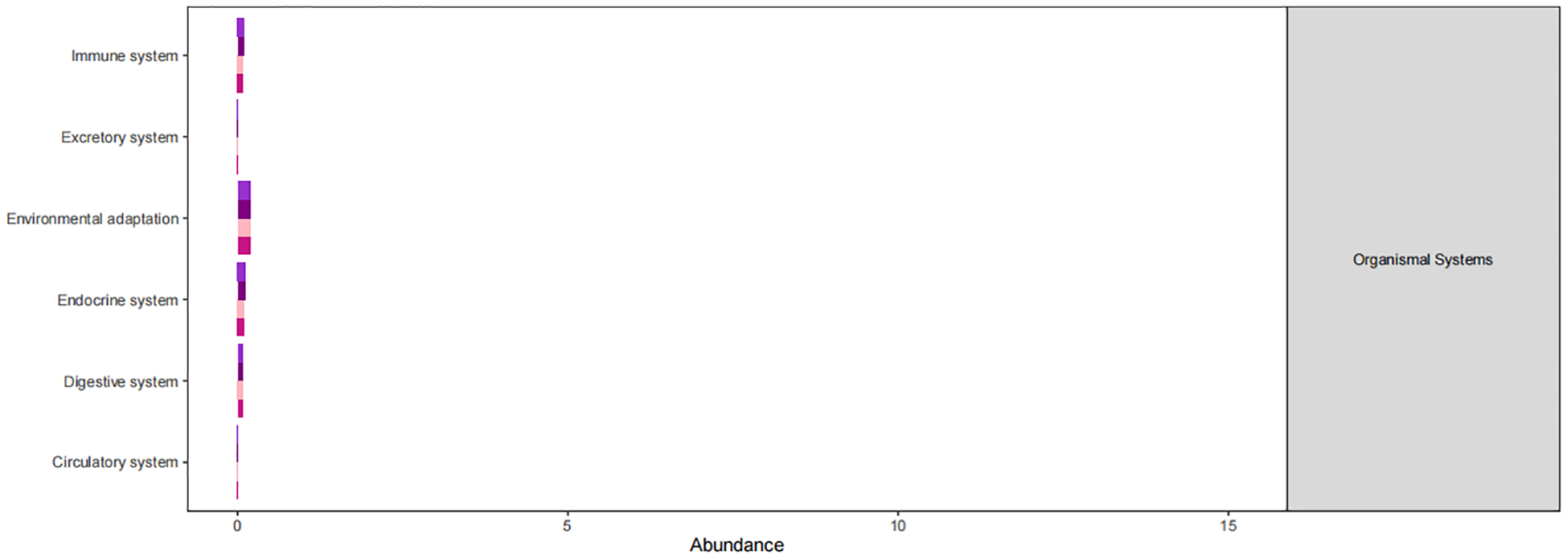
Predicted KEGG functional pathway abundance map (horizontal coordinates: abundance of functional pathways, vertical coordinates: functional pathways at the second classification level of KEGG).

The KEGG database serves as a comprehensive biological metabolic pathway analysis tool, organizing pathways into six main classes: metabolism, genetic information processing, environmental information processing, cellular processes, organismal systems, and human diseases. Each class is further divided into four levels. This study visualizes the impact of average pathway abundance by assessing secondary functional pathways within the KEGG database (**Figures 11**–**14**). Analysis reveals that all samples exhibit higher abundance of bacteria in secondary pathways like amino acid metabolism, carbohydrate metabolism, metabolism of cofactors and vitamins, and lipid metabolism at the first level of genetic information processing and metabolic pathways. These findings could provide valuable insights for future research on gut flora within these high abundance metabolic pathways.

**Figures 11**–**14** Predicted KEGG functional pathway abundance map (horizontal coordinates: abundance of functional pathways, vertical coordinates: functional pathways at the second classification level of KEGG).

## 4 Discussion

With the rapid advancement of modern radiation therapy technology, the number of patients undergoing radiation therapy has steadily risen (10), leading to an increase in associated complications while also enhancing patient survival rates. Among the most common complications is intestinal mucositis, which arises from damage to normal intestinal epithelial cells, resulting in intestinal mucosal atrophy and ulceration that hinders the renewal of underlying epithelial cells, known as radiolucent enteritis (11). Clinically, this condition is classified into acute and chronic radiation enteritis based on its onset time, with this study specifically focusing on acute radiation enteritis. The impact of this condition on patients’ quality of life and radiotherapy regimen is significant, causing inconvenience and serious implications. Despite receiving the same radiation dose, some patients experience radiation enteritis while others show minimal symptoms. Healthcare providers often find themselves reacting passively, only providing symptomatic treatment after the onset of radiation enteritis. However, the current understanding of the mechanisms underlying radiation enteritis remains limited, making it challenging to prevent and manage this condition effectively. Therefore, investigating the etiology of radiation enteritis and identifying preventive measures are pressing issues. The findings presented in this study suggest a correlation between individual fecal microbial profiles and the development of radiation enteritis.

This study conducted a comparative analysis of the intestinal flora structure in four groups of samples before and after radiotherapy, revealing significant differences in flora abundance among the groups. The variations observed could have implications for the effectiveness of radiotherapy and patient prognosis. Specifically, an increase in f:Ruminococcaceae, o:Oscillospirales and g:Faecalibacterium abundance and a significant decrease in the abundance of c:Bacilli were noted in the RE group. while in the patients in the no-RE group, there was a significant increase in the abundance of c:Bacilli in the samples after radiotherapy, which was a statistically significant difference. Wang et al. (12) conducted a study on patients undergoing radiotherapy for cervical cancer, revealing dysbiosis in patients with radiation enteritis, characterized by an increase in Aspergillus and a decrease in Anaplasma. Similarly, Mitra et al. (13) investigated the relationship between changes in intestinal flora and gastrointestinal toxicity in cervical cancer patients undergoing radiotherapy, finding that patients with improved gastrointestinal function had greater diversity in their intestinal flora. These findings suggest that intestinal flora could serve as a potential predictive marker for radiation enteritis. A large clinical trial (14) found that patients undergoing pelvic radiotherapy experienced a decrease in intestinal flora diversity, which was associated with advanced radiation enteritis. The abundance of Clostridium perfringens type IV, Roseobacter, and Vibrio spp. was higher in patients with radiation enteritis, while levels of cytokines like IL-7, IL-12, IL-15, and IL-16, which regulate intestinal flora and homeostasis, were reduced. Furthermore, fecal transplantation treatment led to improvements in clinical symptoms, endoscopic findings, and imaging results in patients with radiation enteritis (15). Although the sample size in these trials was small, the data suggest a potential connection between changes in intestinal flora and radiation enteritis following radiotherapy.

Some animal experimental data suggest that radiation enteritis is associated with changes in intestinal flora. Guo et al. (16) observed an increase in Trichoderma and Enterococcaceae in surviving mice following high doses of radiation, leading to the production of SCFA that helped protect the mice from radiation-induced DNA damage and release of reactive oxygen species (ROS) in hematopoietic and gastrointestinal tissues. Conversely, Gerassy et al. (17) showed that pelvic radiation led to dysbiosis of intestinal flora in a mouse model of rectal radiation, making the intestinal mucosa more vulnerable to radiation damage through the promotion of IL-1β secretion. The impact of intestinal flora on radiation injury was found to be influenced by sex differences, with simvastatin administration mitigating radiation-induced changes in male mice but not in female mice. This suggests that sex-specific intestinal flora plays a crucial role in the treatment of radiation injury (18).

Furthermore, analysis of the functional pathways in the KEGG database revealed high abundance of bacteriophages in secondary pathways like amino acid metabolism, carbohydrate metabolism, cofactors and vitamins metabolism, and lipid metabolism across all samples. These metabolic pathways with high abundance could be closely linked to the changes in the intestinal microenvironment caused by radiotherapy, offering new insights and avenues for future research. These high-abundance metabolic pathways could be intricately linked to the damage and repair mechanisms of intestinal cells induced by radiotherapy. Radiotherapy causes destruction of cells and tissues in the gastrointestinal tract, resulting in inflammation, dysfunction, and disruptions in the balance of gut microbiota. These alterations may consequently impact metabolic processes in the gut, particularly affecting the metabolism of amino acids, carbohydrates, cofactors and vitamins, and lipids. The gut flora plays a significant role in influencing various aspects of radiation damage and repair of the intestinal mucosa. Radiation disrupts the intestinal barrier and mucus layer, resulting in bacterial translocation and activation of the inflammatory response. Radiation-induced dysbiosis can trigger local and systemic immune responses in the intestinal tract, contributing to the development of enterocolitis (8). Intestinal mucosal repair depends on the proliferation and differentiation of intestinal stem cells, which can be regulated by the intestinal microbiota to generate regenerative epithelial cells. Research indicates that bacteria help protect intestinal stem cells from injury by activating the RIG-I/MAVS and STING signaling pathways to induce type I interferon production (19). Furthermore, lipopolysaccharides in Gram-negative bacteria membrane components can stimulate TLR4 expression on epithelial cells, leading to prostaglandin production and reducing radiation-induced apoptosis of epithelial stem cells (20). Key microorganisms and metabolites in high-abundance metabolic pathways could serve as potential targets for treating radiation enteritis. Exploring the functions and mechanisms of these entities can enhance our understanding of their roles in the gut environment, leading to the development of innovative therapeutic strategies. These insights may offer new approaches to restore the gut environment post-radiotherapy and prevent radiation enteritis. By modulating the balance of gut flora and metabolic processes, we may support the recovery and stability of the gut environment, thereby mitigating radiotherapy side effects and enhancing treatment outcomes. Furthermore, these research concepts could be extrapolated to other diseases linked to the gut environment, highlighting the importance of studying intestinal microenvironment changes post-radiotherapy for informing novel therapeutic interventions in related conditions.

The study is limited by a small sample size consisting solely of patients with acute radiation enteritis. There is a lack of follow-up to analyze the correlation with chronic radiation enteritis and intestinal flora. Factors such as stage diet, geographic location, and nutrient preparation therapy were not considered. Additionally, the study did not include analysis of fecal characteristics and the frequency of excretion in specimen collection to enhance data correlation. Furthermore, only fecal specimens before and during radiation therapy were analyzed, with no post-radiotherapy specimens included. The study solely relied on 16SrRNA sequencing to identify differences in intestinal flora between patients with and without radiation enteritis during radiotherapy, lacking metabolomics analysis and macro-genome sequencing. Future research would benefit from a large sample size, dynamic, multistage approach. This study excluded patients who had used antibiotics within 1 month prior to radiotherapy. However, in the clinical management of pelvic malignant tumors, antibiotics are routinely used in surgical treatment to prevent postoperative infections. Additionally, a significant number of patients require postoperative radiotherapy. Some studies have indicated that pretreatment with antibiotics in mice can effectively mitigate radiation-induced intestinal flora disorders and damage. Therefore, future research should consider including this patient population in relevant discussions. In conclusion, there is a need for comprehensive studies with large sample sizes, conducted at multiple stages and on a genome-wide level, to explore the mechanisms of action in depth. This will help advance microbial therapy as a leading treatment for radiolucent enterocolitis and provide valuable insights for the prevention and treatment of this condition in clinical settings.

## Data availability statement

The raw data supporting the conclusions of this article will be made available by the authors, without undue reservation.

## Ethics statement

The study was approved by the Ethics Committee of Qingdao Hiser Hospital Affiliated of Qingdao University (Qingdao Traditional Chinese Medicine Hospital). Ethical Approval Number: 2021HC01LS022. The studies were conducted in accordance with the local legislation and institutional requirements. The participants provided their written informed consent to participate in this study. Written informed consent was obtained from the individual(s) for the publication of any potentially identifiable images or data included in this article.

## Author contributions

LL: Conceptualization, Writing – original draft. YZ: Conceptualization, Writing – original draft. JZ: Conceptualization, Methodology, Writing – review & editing. PLiu: Formal analysis, Writing – review & editing. WL: Formal analysis, Writing – review & editing. CS: Formal analysis, Writing – review & editing. DT: Formal analysis, Writing – review & editing. PLi: Formal analysis, Writing – review & editing. JT: Conceptualization, Data curation, Methodology, Writing – review & editing. JX: Conceptualization, Funding acquisition, Project administration, Writing – review & editing.
